# Bowel obstruction secondary to gallstone ileus within an inguinoscrotal hernia: a rare diagnosis in an elderly patient

**DOI:** 10.1259/bjrcr.20200207

**Published:** 2021-03-11

**Authors:** Nisham Ghimire, Diogo JV Silva, Akshay Bavikatte, Mojolaoluwa Olugbemi, Ami Mishra, Sarah Ann Smith

**Affiliations:** 1 West Suffolk Hospital, Suffolk, UK

## Abstract

Gallstone ileus and obstructed inguinal hernias are respectively, rare and common causes of small bowel obstruction. There are no published cases of these pathologies occurring simultaneously. Here, we describe a unique case of an elderly male patient presenting with a small bowel obstruction caused by these combined pathologies. Following an acute presentation with obstructive symptoms, a CT scan demonstrated small bowel obstruction due to a large gallstone lodged in the neck of an inguinoscrotal hernia with associated pneumobilia. The case may have been managed conservatively if it was not for the presence of the gallstone. Previous imaging had incidentally demonstrated gallstones in the gallbladder and a large uncomplicated right inguinoscrotal hernia. It is presumed that a cholecystoduodenal fistula formed and a gallstone then migrated downstream to lodge within the neck of the inguinoscrotal hernia. This case underscores the concept that even in the presence of an “obvious” cause of small bowel obstruction, such as an irreducible, large inguinoscrotal hernia, we must always maintain a healthy clinical skepticism and an open mind to other unexpected aetiologies, which may account for the clinical presentation that might impact subsequent management.

## Background

Inguinal hernias are common with a lifetime risk of 27–43% in males and 3–6% in females^1^ and account for 20% of small bowel obstructions.^2^ In contrast to that, gallstone ileus is responsible for just less than 1%.^3^ Small bowel obstruction related to gallstone ileus within an inguinal hernia is a much rarer presentation with no previous documentation in the literature. Following cholecystitis, adhesions may form between the gallbladder and the duodenum and, with associated pressure from a gallstone, a cholecystoduodenal fistula can form. A gallstone may then proceed through the intestinal tract until it reaches an area of narrowed lumen, usually the distal ileum or a fixed obstruction point, such as an inguinoscrotal hernia in this case. Delayed diagnosis, due to atypical or misleading symptoms such as constipation may lead to further complications, especially when associated with synchronous pathologies.^4^ We discuss a rare case with no prior description in the medical literature, where the abdominal CT scan was essential in establishing the diagnosis. It highlights that past medical history and pre-existing imaging can provide important clues to aid an accurate diagnosis and execute appropriate management.

## Case presentation

A 93-year-old gentleman was brought in by ambulance to our emergency department with a 24-h history of abdominal pain and distension associated with one episode of vomiting. The patient was known to have a longstanding large right-sided inguinoscrotal hernia. Additionally, he had co-morbidities of asthma, chronic renal failure and a long-term urinary catheter for benign prostatic hyperplasia. On examination, the abdomen was soft but distended and a large irreducible right-sided inguinoscrotal hernia was noted.

## Investigations

**Figure 1.**
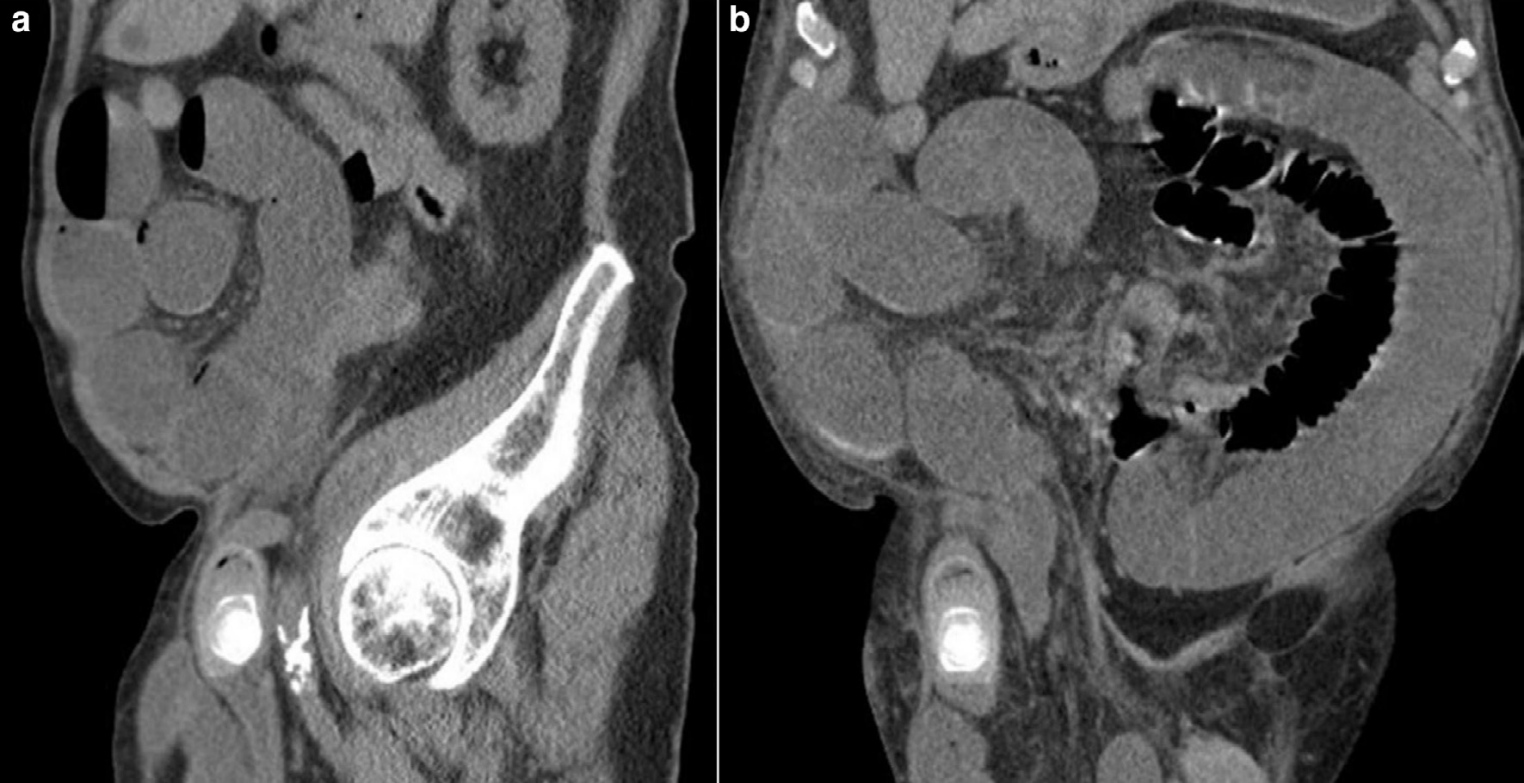
(a, b) Sagittal and coronal sections of the presentation unenhanced CT study showing a large gallstone lodged at the neck of the right inguinoscrotal hernia. Dilated loops of upstream small bowel are demonstrated within the abdominal cavity and within the hernial sac.

**Figure 2.**
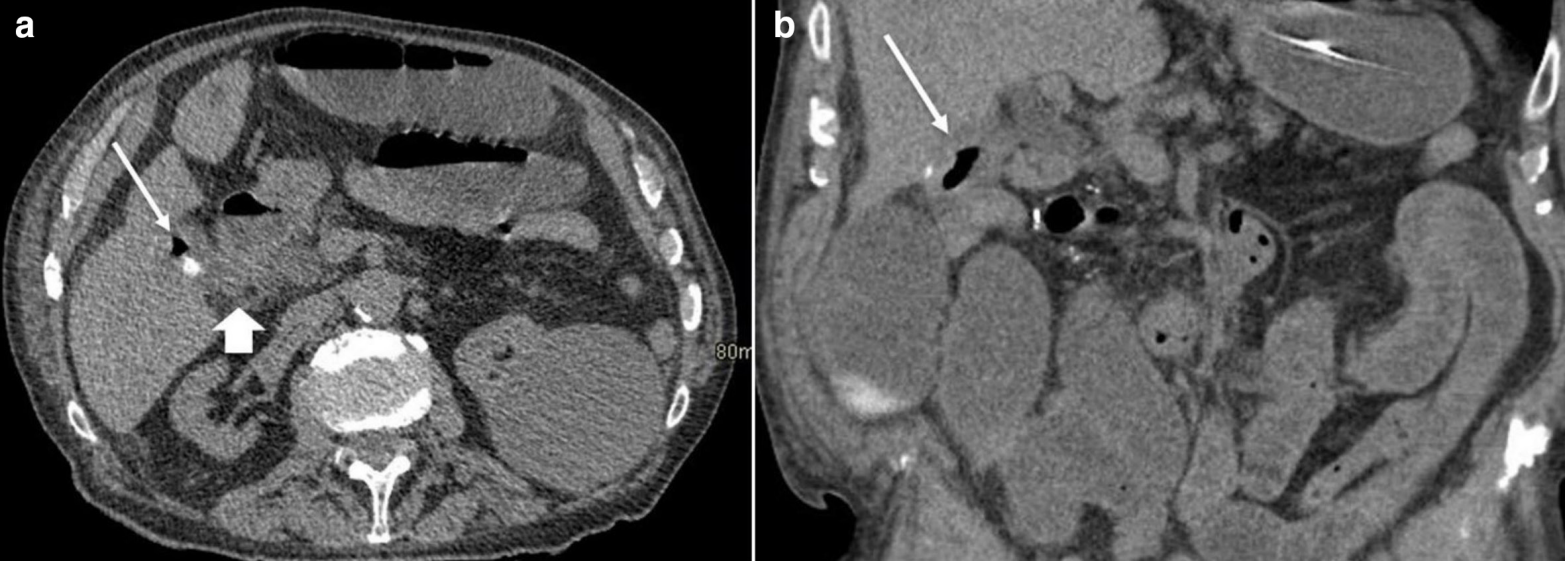
(a, b) Coronal and axial sections at the level of the upper abdomen on the presentation CT, demonstrating gas and a small calcified gallstone within a thick-walled gallbladder (arrowed). Also noted are dilated small bowel loops. There is loss of fat plane between the gallbladder and the second part of the duodenum with surrounding fat stranding suggestive of the site of cholecystoduodenal fistula (arrow head).

**Figure 3.**
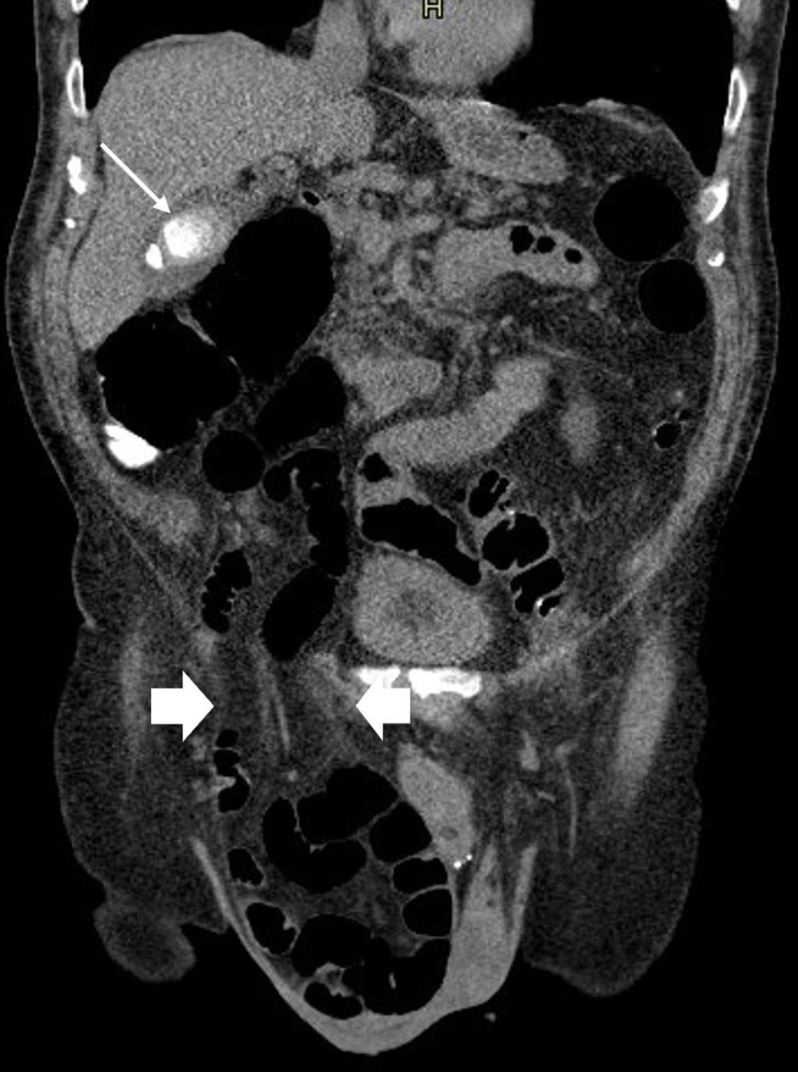
Coronal section of the previous unenhanced CT colonoscopy which demonstrated large and small calcified gallstones in the gallbladder (thin arrow) and a large right inguinoscrotal hernia containing small bowel loops (arrow heads delineating the neck).

An abdominal radiograph showed distended gas-filled loops of small bowel. Intravenous fluids were administered and a nasogastric tube was inserted. The renal function was compromised with an eGFR of 26 mL/min/1.73 m^2^ on a background of chronic kidney disease. Therefore, a non-contrast CT scan of the abdomen and pelvis was performed to further investigate his obstructive symptoms. The CT scan showed dilated small bowel loops to a transition point within the right inguinoscrotal hernia where a 42 mm calcified stone was lodged at the neck (Figure 1a and b). Downstream loops of small bowel were collapsed. Gas was noted to be present in the thick-walled gallbladder which also contained small calcified stones (Figure 2a). There was loss of fat plane between the gallbladder and the second part of the duodenum with surrounding fat stranding, suggestive of the site of a cholecystoduodenal fistula (Figure 2b). The patient had had a previous CT colonoscopy performed 4 years earlier to investigate weight loss and change in bowel habit, a cause for which was not found, but which incidentally noted cholecystolithiasis and a right uncomplicated inguinoscrotal hernia containing small bowel loops (Figure 3). Even in retrospect, neither the obstructing gallstone nor the pneumobilia could be identified on the plain film. On the basis of history, clinical features and the radiological findings, the diagnosis of small bowel obstruction caused by a gallstone lodged in a right inguinoscrotal hernia was established.

## Treatment

**Figure 4.**
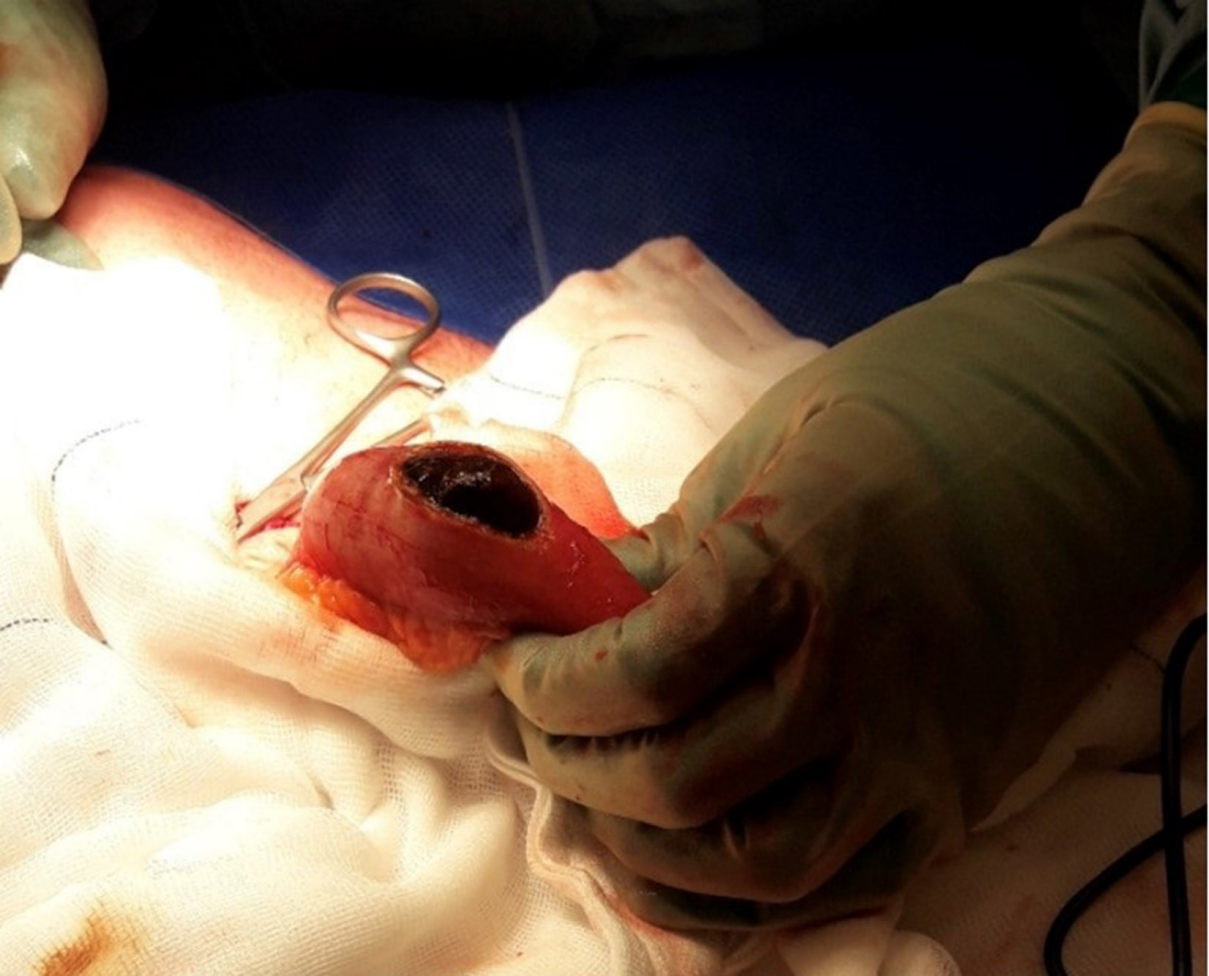
Intraoperative image of the enterolithotomy – the large gallstone was removed from the herniated small bowel loop.

A discussion with the patient and his family regarding further management was undertaken, emphasising concerns about his age and associated comorbidities, as well as disclosing the high mortality risk of 23.5%, (based on the UK National Emergency Laparotomy Audit (NELA) risk adjustment model). A right groin exploration was performed, and the obstructing gallstone was identified within the small bowel. An enterolithotomy (Figure 4) (without concurrent cholecystectomy) was performed via the groin incision, the hernia was reduced, and an orchidectomy was performed as planned pre-operatively to facilitate repair. The inguinal canal was reinforced with a biological mesh (Phasix^TM^ Mesh^®^) and the wound was closed.

## Outcome and follow-up

The patient had a good recovery after the operation and was discharged to the community with a plan for follow up in 6 months. He developed a minor surgical site infection 3 weeks post-operatively which was managed with antibiotics.

## Discussion

Small bowel obstruction is one of the most common surgical emergencies seen in elderly populations, accounting for 51% of all emergency laparotomies in the UK.^5^ The most common cause of small bowel obstruction is adhesions (>70%) and less commonly hernias and strictures.^6^ Gallstone ileus is thought to be the underlying aetiology of 1–4% of hospital presentations with intestinal obstruction and for up to 25% of all non-strangulated cases of small bowel obstruction in patients over 65 years of age.^7^ Inguinal hernias account for up to three-quarters of abdominal wall hernias and may present with obstruction.^8^ In the case we present, the patient had features of small bowel obstruction secondary to a gallstone lodged within the neck of an inguinoscrotal hernia which makes it interesting and unique within the literature.

Following cholecystitis, adhesions may form between the gallbladder and the adjacent bowel, most frequently the duodenum. Pressure effect from gallstones cause them to erode through and migrate into the bowel forming a cholecystoenteric fistula. The gallstone may then impact in any part of the intestinal tract, most commonly in the ileum (50%–60.5% - where the lumen of the gastrointestinal tract is the narrowest), jejunum (16.1%–26.9%), duodenum (3.5%–14.6%) and colon (3.0%–4.1%).^9^ Ayantunde et al^10^ reported known gallstone disease in only 27% of cases and other case series show that 50% of the patients denied any biliary symptoms.^7^ Although a plain abdominal radiograph is routinely used in the initial assessment of small bowel obstruction, it only has a sensitivity of 40–70% in the diagnosis of gallstone ileus.^11^ To confidently make the diagnosis of gallstone ileus on plain film, a triad of dilated loops of small bowel, pneumobilia and presence of a gallstone is required.^11^ These features are also required on the CT scan, but it offers overall sensitivity, specificity, and accuracy of 93%, 100%, and 99%, respectively.^12^ In this case, the clinical presentation was of straightforward small bowel obstruction supported by the initial plain abdominal radiograph. However, the aetiology for the obstruction was not clearly demonstrated during the initial evaluation, as the hernia was not initially recognised as the site of obstruction, and the patient belongs to the minority who have a known history of asymptomatic gallstones. Although CT is routinely the main tool used to determine the cause of small bowel obstruction, it was of particular use in this unique case. The CT scan, besides providing the diagnosis, guided surgical management by localising the gallstone to the scrotum, allowing a groin incision rather than a midline abdominal incision laparotomy in a high-risk patient.

Since there is a low rate of spontaneous stone passage of approximately 1.3%,^13^ surgery via a midline laparotomy is indicated for most cases of gallstone ileus. There has been debate for some time for the best management for gallstone ileus. Enterolithotomy alone is preferred and sufficient in elderly patients without concurrent cholecystectomy, as this adds considerable risk to the procedure’s mortality.^3,14^

Recent studies have reported safe use of biologic mesh reinforcements in contaminated ventral hernia repair.^15,16^ Our patient underwent open inguinal hernia repair with removal of the gallstone via an enterotomy, that was subsequently closed. The hernia was reduced and following an orchidectomy, the posterior wall of the inguinal canal was reinforced with a biological mesh. The orchidectomy (for which consent had been obtained from the patient) was performed to enable closure of the large defected deep ring, to reduce the risk of a recurrence and is a well-documented approach for repairing large inguinal hernias.^17^ The patient recovered well with no significant post-operative complications.

An exhaustive search of literature and peer reviewed libraries was done, including Cochrane and PubMed Libraries, and did not identify a similar presentation. Even though gallstone ileus within an inguinal hernia is rare, it does exist and this described case emphasises the requirement for pre-operative CT to aid surgical planning. It carries a significant risk of morbidity and mortality; therefore, early diagnosis and treatment is essential for an improved final outcome. The patient’s previous history and prior radiological investigations play a vital role in identifying such conditions. Current radiological findings and consideration of the patient’s co-morbidities guide management. This rare case upholds the importance of the role of CT imaging in the diagnosis and management of a patient with bowel obstruction. Furthermore, early and considered surgical management, especially in an elderly patient with comorbidities, can significantly reduce the rates of morbidity and mortality associated with this pathology.

## Learning points

1. CT imaging is essential in the diagnosis and management of small bowel obstruction, even in the presence of an “obvious” clinical cause. Keep an open mind to an unusual cause or combined pathology.
2. The review of past imaging may be helpful in clinching the diagnosis.
3. Early cross-sectional imaging facilitates early initiation of treatment.
4. CT scanning may improve patient outcome by tailoring surgical approach and technique.
5. Gallstone ileus occurs following migration of a gallstone via a cholecystoenteric fistula to lodge at a point of ileal luminal narrowing.
6. Gallstone ileus is a rare cause of small bowel obstruction. The most common causes are adhesions, hernias and strictures.
7. Gallstone ileus and inguinal hernias may be found as separate causes of small bowel obstruction but the finding of a gallstone ileus within an inguinal hernia is a very rare presentation and indeed could only have been identified pre-operatively by CT. 
